# Comparison of accommodation and accommodative micro-fluctuation after implantable collamer lens and LASIK surgery for myopia

**DOI:** 10.1186/s12886-021-02217-6

**Published:** 2022-01-04

**Authors:** Li Li, Bo Zhang, Zheng Wang

**Affiliations:** 1 Aier School of Ophthalmology, Central South University, Fourth Floor, New Century Mansion, 198 Middle Furong Road, Changsha, China; 2 Department of Refractive Surgery, Guangzhou Aier Eye Hospital, Guangzhou, China; 3 Chongqing Eye and Vision Care Hospital, Chongqing, China; 4 Aier Institute of Refractive Surgery, Aier Eye Hospital Group, Changsha, China

**Keywords:** ICL implantation, LASIK, Accommodation, Accommodative micro-fluctuation

## Abstract

**Background:**

To longitudinally analyze and compare the accommodative micro-fluctuation (MFs) and accommodative function between myopic patients after implantable collamer lens (ICL) implantation and laser in situ keratomileusis (LASIK).

**Methods:**

Patients with good corrected visual acuity (20/20 or better) and underwent ICL (V4c) and LASIK for myopic-correction (ranging from − 3.50 to − 8.50 D) were recruited. Refraction, amplitude of accommodation (AMP), accommodative lag, higher-order aberration (HOA), and MFs were recorded before surgery and 1 and 3 months after surgery. The ACOMEREF automatic refractor was used to measure the high-frequency component (HFC) of the MFs, which suggested tension of the ciliary muscle.

**Results:**

The study comprised 120 eyes. At 3 months after surgery, the manifest refractive spherical equivalent of the ICL and LASIK groups were − 0.11 and − 0.09 D, respectively (*p* = 0.46). HFC values were significantly higher at 1 month (*p* = 0.03) and 3 months postoperatively (*p* = 0.03) in the ICL group compared to that in the LASIK group. The ocular HOA of the ICL group was 1.08 ± 0.43 μm, which was lower than the LASIK group 1.45 ± 0.54 μm (*p* = 0.01). No significant differences in AMP and accommodative lag between groups were noted at 3 months postoperatively. There was a positive correlation between HFC and vault of the ICL lens (*r*^2^ = 0.14, *p* = 0.005). There were no correlations between HFC and ocular HOA and postoperative MRSE in the two groups (all *p>*0.05).

**Conclusions:**

The HFC increased significantly after an early period of ICL implantation compared to laser in situ keratomileusis for myopic correction, which indicated increased tension of the ciliary muscle, and had a positive correlation on the vault of the ICL lens; However, studies with longer follow-up time and more structural evaluation are needed.

## Background

Laser in situ keratomileusis (LASIK) is the dominant and most effective operation for the correction of myopia [1–3]. However, after LASIK surgery, especially in the early period, there have been some visual complaints, such as fatigue or blurriness at near-distance working, which may be responsible for accommodative dysfunction or increased accommodative need for near-working [4]. Implantable collamer lens installation (ICL) is becoming an increasingly acceptable treatment method for myopia [5]. Previous studies [6–8] showed the safety and effectiveness of ICL correction in myopic eyes. Outcomes from these studies have been dedicated to the viability of ICL as an alternative way to current corneal refractive surgery. Previous publications [9, 10] have demonstrated that visual quality, such as higher-order aberration (HOA) and contrast sensitivity after ICL, was better than in eyes that had LASIK. Until now, there was still no research comparing the accommodative function of the two methods. Moreover, there are still controversies about accommodative function after ICL implantation [11, 12].

Accommodative micro-fluctuations (MFs) are one of the important parameters for evaluating accommodative function. MFs refer to a state under a stable accommodative stimuli when real-time accommodative power of the human eye fluctuates within a certain range [13]. Moreover, one previous study showed that high-frequency (1.3 to 2.2 Hz, HFC) of MFs represent tension of the ciliary muscle, that is, when the ciliary muscle contraction load increases, the HFC increases [14]. The ACOMEREF refractometer (Righton, Japan), which uses infrared light to record the HFC of MFs under different accommodative stimuli, has been proven to be useful to objectively evaluate the stress of the ciliary muscle [15].

The purpose of this study was to analyze and compare the subjective accommodation and objective accommodative micro-fluctuations between patients after ICL and LASIK, and to identify possible factors that impact accommodation in the two groups, such as refraction, aberration, or ICL implantation.

## Methods

### Patients and study design

This prospective, and case-controlled study included 60 eyes from 30 patients who were scheduled for implantation of the V4c Visian ICL (STAAR Surgical Company, Monrovia, California, USA), and 60 eyes from 30 patients who were scheduled for LASIK to correct myopia or myopic astigmatism with refraction ± spherical equivalent − 6.13 ± 1.17 D (range − 3.50 to − 8.50 D). Both surgical treatments were performed by one surgeon (Dr. Wang). The study conformed with the tests of the Declaration of Helsinki, and prior informed consent was obtained from all participants. This study was approved by the Ethics Committee of Guangzhou Aier Eye Hospital.

Patient were included in this study if their age was over 21 years, manifest refractive spherical equivalent (MRSE) had maintained stability (the increase was less than 0.5 D per year) for more than 2 years, and their best corrected distance visual acuity was better than 20/20.

Patient were excluded from this study if their age was older than 40 years, they had previous ocular surgery, density of the corneal endothelial cell was ≤2000 cells/mm^2^, depth of the anterior chamber was ≤2.8 mm, and if there was evidence of corneal infection, corneal inflammation, glaucoma, amblyopia, anisometropia, presbyopia, keratoconus, or retinal detachment.

### LASIK and ICL procedures

#### LASIK procedure

The FS200 (Alcon Laboratories, Ft Worth, TX, USA) was used to create a corneal flap of thickness 110 to 120 μm. The Wavelight EX500 (Alcon Laboratories) with custom-Q mode was used to correct myopia or myopic astigmatism with a 6.5 mm diameter optical zone. The refraction was adjusted according to a similar nomogram recommendation (A_LI_D1_Nomogramm STD_10_2007 Rev. 0 Mar 2011).

#### ICL implantation

The V4c ICL was inserted through a 2.8 mm corneal incision within the anterior chamber preserving viscosurgical material (Opegan; Santen, Osaka, Japan). The implanted ICL was then moved from the anterior chamber to the posterior chamber. After the ICL was lifted to the posterior chamber, the viscosurgical material was completely washed out using a balanced saline solution. Manifest refraction (without nomogram adjustment) was used to calculate the lens power performed by STAAR, and all eyes were targeted for emmetropia.

#### Measurement

The UCVA, near vision (40 cm), MRSE, aberration, accommodative function, and MFs were recorded before the operation, and 1 month and 3 months postoperatively. The root mean square of the spherical-like aberration (12th = ${Z}_4^0$ and 24th = ${Z}_6^0$ terms), coma-like aberration (7th = ${Z}_3^{-1}$, 8th = ${Z}_3^1$, 17th = ${Z}_5^{-1}$, and 18th = ${Z}_5^1$ terms) and trefoil-like aberration (6th = ${Z}_3^{-3}$, 9th = ${Z}_3^3$, 16th = ${Z}_5^{-3}$, and 19th = ${Z}_5^3$ terms) of the ocular, cornea, and internal aberration were tested by OPD-Scan®III (NIDEK, Japan) for 6-mm pupils. Total HOA were calculated as the root mean square of all terms including the third, fourth, fifth, and sixth order. The accommodative function included the amplitude of accommodation (AMP) and accommodative lag. The AMP was measured using the minus lens method, and the accommodative lag were measured using the fusion cross-cylinder method based on previous study [11]. The AMP, accommodative lag, and the MFs parameters were required to be measured three times, and the average value was taken as the final outcomes. At 1 month and 3 months postoperatively, we tested the central ICL vault by the anterior segment optical coherence tomography (in a similar darkness conditions.

#### Accommodative Micro-fluctuation

The ACOMOREF 2 (Righton, Japan) with AMF mode was used to record the MFs outcomes in this study [15]. It is an infrared optometer with a spectral power calculation to analysis and record the non-stationary spectrum of MF. The MFs were caused by the movement of the crystalline lens due to ciliary muscle oscillation under accommodative stimuli. Before starting the examination, patients were asked to relax in a dark room for at least 5 min, and the MFs measurement was tested on the dominant eye first. During the testing period, the patients were requested to stare clearly at the fixation target with minimal and quick blinks. The accommodative stimuli were set from + 0.50 D to − 3.00 D in 0.50 D increments that included eight accommodative stimuli. The waveforms of accommodative responses were transformed into a three-dimension graph, which demonstrates HFC, accommodation stimulus, and amplitude of accommodative response.

### Statistical analysis

Statistical analysis was performed using SPSS software (version 22.0, International Business Machines Corp). *P*-values less than 0.05 were considered statistically significant. Data were expressed as the mean ± standard deviation. The Kolmogorov–Smirnov test was used for confirming data normality, independent t-tests for continuous variables, and Wilcoxon tests for comparing continuous variables without normality.

## Results

A total of 60 people (120 eyes) participated in this study with an average age of 27.6 ± 4.9 years (range 20–38 years). As shown in Table 1, the preoperative baseline parameters of groups were well-balanced, including age, MRSE, AMP, accommodative lag, HFC, and HOA (all *p*>0.05). At 3 months after surgery, there was no significant difference in postoperative MRSE between the two groups (− 0.11 ± 0.24 D for the ICL group versus − 0.09 ± 0.18 D for the LASIK group, *p* = 0.46).

**Table 1:** The preoperative baseline characteristics of the ICL and LASIK groups

Parameter		ICL Group (*n* = 60 eyes)	LASIK Group (*n* = 60 eyes)	t	*P* value
Age		28.2 ± 4.1	27.0 ± 5.6	0.66	0.64
Sphere		−5.67 ± 1.25	−5.78 ± 0.92	0.34	0.75
Cylinder		−0.74 ± 0.86	−0.86 ± 0.63	0.62	0.74
Spherical equivalent refraction		−6.05 ± 1.21	−6.21 ± 1.13	0.51	0.69
Amplitude of accommodation		4.33 ± 0.86	4.48 ± 0.84	0.67	0.51
Accommodative lag		0.80 ± 0.44	0.50 ± 0.44	1.83	0.08
HFC		59.31 ± 2.33	59.27 ± 2.11	0.08	0.94
Ocular	Total HOA	7.05 ± 1.58	7.66 ± 1.33	1.52	0.14
	Coma	0.19 ± 0.09	0.16 ± 0.08	0.88	0.38
	Trefoil	0.25 ± 0.12	0.22 ± 0.09	1.02	0.31
	SA	0.09 ± 0.06	0.09 ± 0.07	0.07	0.95

*ICL* Phakic posterior chamber implantable contact lens implantation (V4c), *LASIK* Laser in situ keratomileusis, *HFC* High-frequency of accommodative micro-fluctuations, *HOA* Higher order aberration, *SA* Spherical aberrations, *t* Student’s t-test

### AMP and accommodative lag

**Fig. 1:**
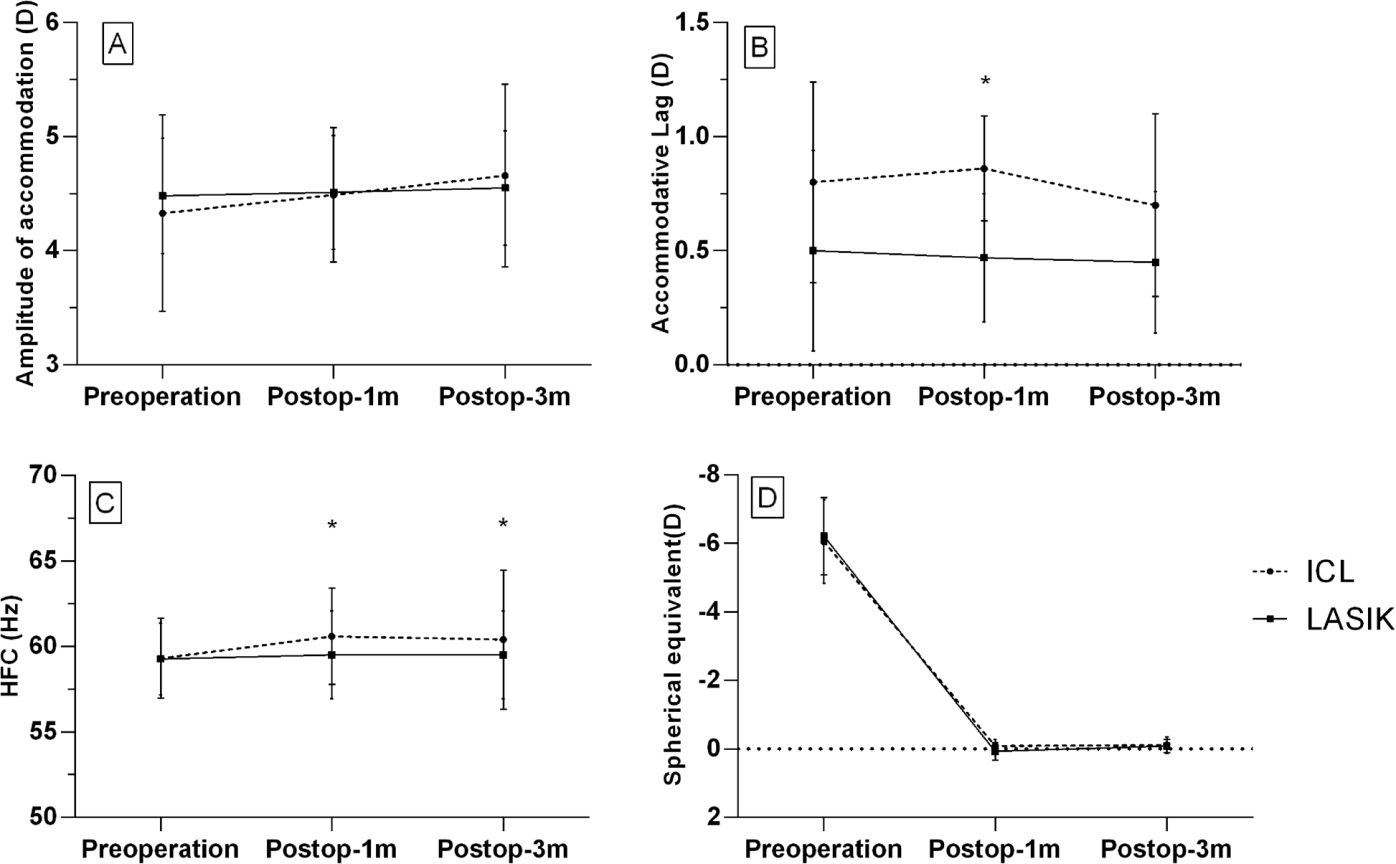
Preoperative and postoperative amplitude of accommodation (**A**), accommodative lag (**B**), HFC (**C**) and refraction (**D**) after ICL and LASIK surgeries. (Postop-1 m means 1 month after operation; Postop-3 m means 3 months after operation; HFC means high-frequency component of the accommodative micro-fluctuation; * indicates that there was a statistically significant difference between the two groups by student-t test)

As shown in Fig. 1, the accommodative lag of the ICL group were higher than the LASIK group (0.86 ± 0.23 D vs. 0.47 ± 0.28 D, *p<*0.05) at 1 month after surgery. There was no significant difference in the accommodative lag between the ICL and the LASIK groups at 3 months after surgery. The accommodative lag of the ICL group showed an early increasing and later decreasing trend in the 1- to 3-month follow-up period. However, in the LASIK group, accommodative lag showed a continuously decreasing trend. The differences in the changes of accommodative lag (postoperative versus preoperative) between the two groups at 3 months postoperatively were not statistically significant (*p>*0.05). At 3 months after surgery, there were no differences in the AMP values in both ICL and LASIK surgeries (all *p>*0.05). The postoperative AMP of the ICL and the LASIK group was 4.66 ± 1.04 D and 4.55 ± 1.39 D, respectively (*p* = 0.43). There was no difference in the postoperative AMP and Δ AMP of two groups at 1 and 3 months after surgery (all *p>*0.05).

### Higher-order Wavefront aberration

**Fig. 2:**
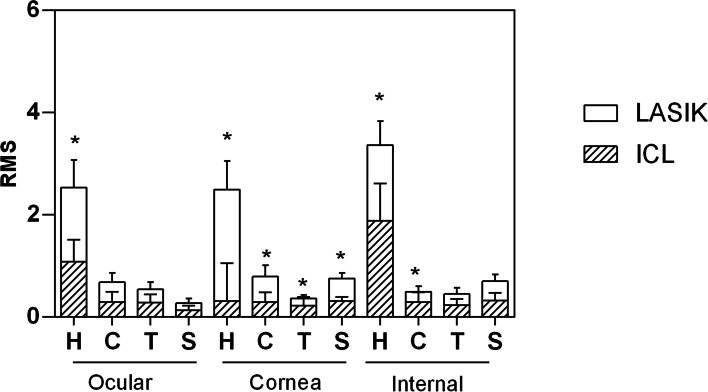
The ocular, corneal, and internal HOAin eyes undergoing implantable collamer lens implantation (V4c) and laser in situ keratomileusis for 6-mm pupils at three months postoperatively. (HOA: Higher order aberration; H: Total HOA; C: Coma; T: Trefoil; S: Spherical aberration)

As shown in Table 2, the ocular Δcoma, corneal Δ spherical aberration (SA), corneal Δcoma, and internal ΔSA that were induced were significantly lower after ICL implantation than after LASIK (*p* = 0.01, < 0.001, < 0.001, and 0.02, respectively). At 3 months after operation, the ocular total HOA of the ICL group was 1.08 ± 0.43 μm, which was lower than the LASIK group 1.45 ± 0.54 μm (*p* = 0.01). The corneal HOA, coma, and SA of the ICL group were 0.31 ± 0.74 μm, 0.29 ± 0.19 μm, and 0.31 ± 0.08 μm, which were lower than those of the LASIK group (all *p* < 0.05) (Fig. 2).

**Table 2:** Higher-order aberration in eyes undergoing phakic posterior chamber implantable contact lens implantation (V4c) and laser in situ keratomileusis for 6-mm pupils at three months postoperatively

Parameters	ICL Group	LASIK Group	t	*P* value
**Ocular**				
Total HOA changing (Δ total HOA)	6.21 ± 1.43	5.67 ± 2.23	1.05	0.30
Coma changing (Δ Coma)	−0.10 ± 0.16	− 0.24 ± 0.19	2.81	0.01
Trefoil changing (Δ Trefoil)	−0.04 ± 0.13	− 0.05 ± 0.12	0.34	0.73
SA changing (Δ SA)	−0.04 ± 0.14	−0.05 ± 0.13	0.31	0.75
**Corneal**				
Total HOA changing (Δ total HOA)	−0.20 ± 0.51	−0.49 ± 0.77	1.66	0.09
Coma changing (Δ Coma-like aberrations)	0.08 ± 0.10	−0.32 ± 0.25	6.36	<.001
Trefoil changing (Δ Trefoil-like aberrations)	−0.05 ± 0.14	− 0.04 ± 0.10	0.39	0.69
SA changing (Δ Spherical aberration)	−0.02 ± 0.05	− 0.17 ± 0.11	0.99	<.001
**Internal**				
Total HOA changing (Δ total HOA)	4.30 ± 0.15	4.75 ± 2.30	0.86	0.40
Coma changing (Δ Coma)	−0.07 ± 0.17	−0.04 ± 0.13	0.73	0.46
Trefoil changing (Δ Trefoil)	−0.05 ± 0.13	−0.07 ± 0.15	1.00	0.67
SA changing (Δ SA)	−0.01 ± 0.10	−0.09 ± 0.14	0.63	0.02

*ICL* Phakic posterior chamber implantable contact lens implantation (V4c), *LASIK* Laser in situ keratomileusis, *HOA* Higher order aberration, *SA* Spherical aberration, Δ: The difference between preoperative and postoperative values; t: Student’s t-test

### Micro-fluctuations

**Fig. 3:**
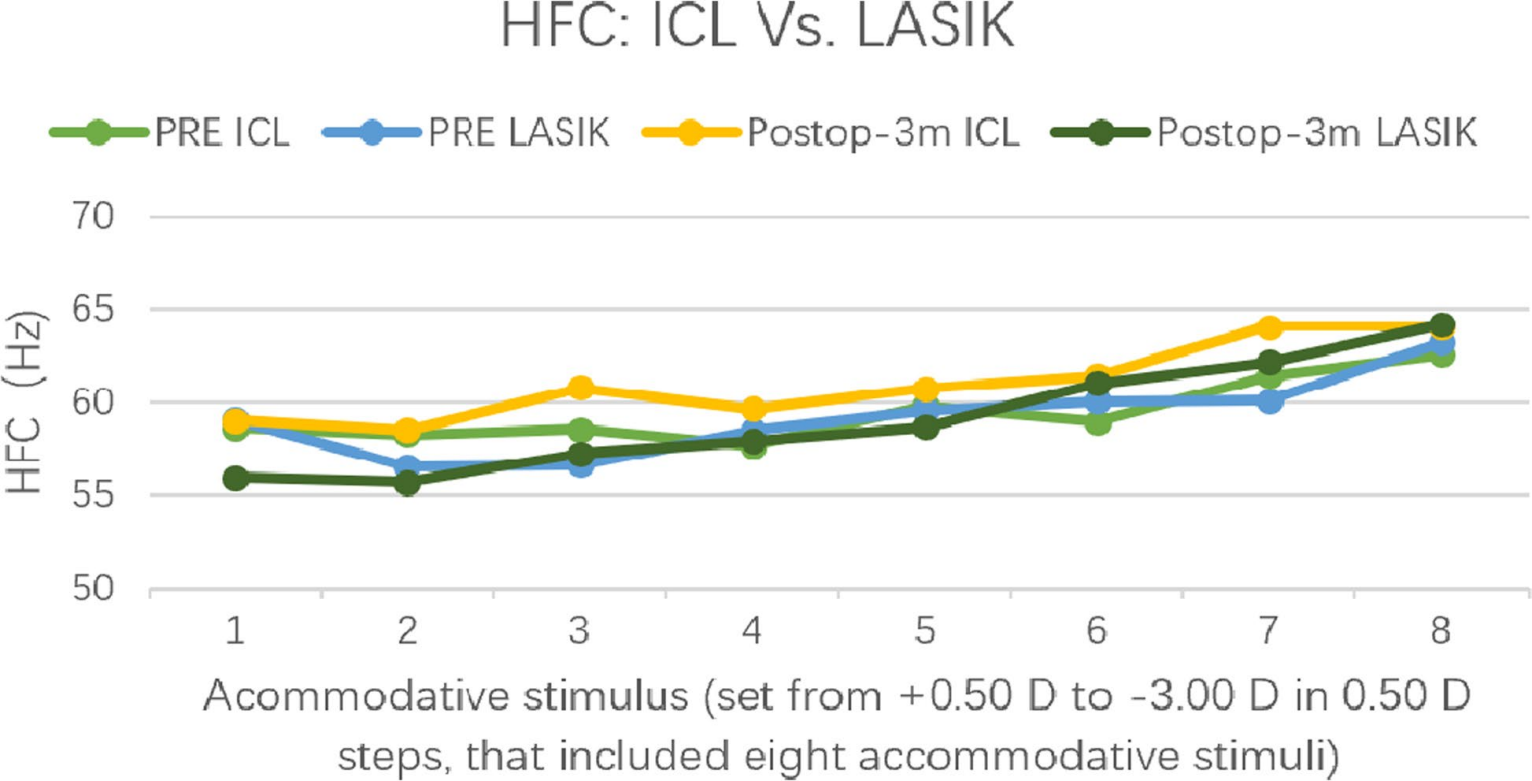
The HFC outcomes within different accommodative stimuli between LASIK and ICL groups before and 3 months after surgery (HFC means high-frequency component of the accommodative micro-fluctuation)

In the ICL group, the HFC at 1 month and 3 months after surgery was 60.60 ± 2.82 and 60.40 ± 4.07 Hz, respectively, which were significantly increased compared with the preoperative HFC (*p* = 0.04 and 0.03, respectively). In the LASIK group, there was no significant differences between the postoperative and the preoperative HFC at any follow-up timepoint, and the HFC values at 3 months after surgery were 59.51 ± 2.56 Hz. The HFC of the ICL group was higher than that of the LASIK group at 1 month (*p* = 0.03) and 3 months (*p* = 0.04) after surgery. As shown in Fig. 3, at 3 months after the operation, with increased accommodative stimulus in the ICL group, the amplitude of HFC also increased; the HFCs of the ICL group under eight different accommodative stimuli were higher than those of the LASIK group.

### Correlation: HFC versus MRSE, Δ HOA and Vault

**Fig. 4:**
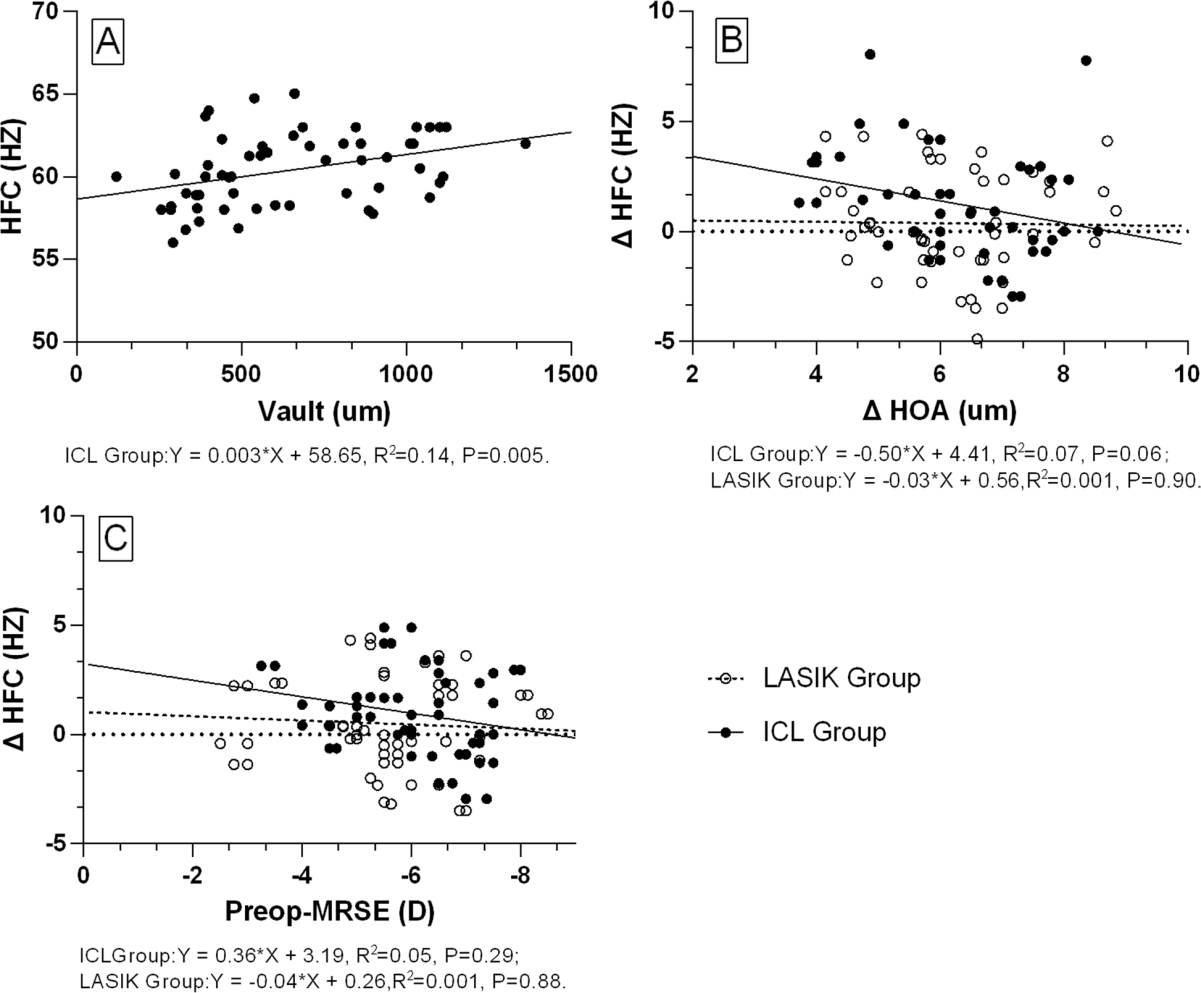
Linear regression analysis demonstrating the relationships between postoperative HFC and **A**) vault of implantable collamer lens, **B**) ΔHOA (preoperative- vs. postoperative-ocular total higher-order wavefront aberration for 6 mm pupils), and **C**) preoperative manifest refractive spherical equivalent in ICL and LASIK groups. Postoperative HFC had a correlation with the vault of ICL (*p<*0.05)

As shown in Fig. 4, at 3 months after the operation, there were no correlations between HFC and ocular Δ total HOA and postoperative MRSE in the two groups (all *p>*0.05). In addition, in the ICL group, there was a relationship between the postoperative HFC and the vault (*r*^2^ = 0.14, *p* = 0.005, *Y* = 0.003*X + 58.65), which indicated that as the vault increased, the HFC value increased.

## Discussion

Our study showed that the V4c Visian ICL had comparable objective accommodation compared to LASIK surgery for myopic correction, within similar AMP and accommodative lag outcomes. The HFC increased significantly after 3 months of ICL implantation, which represent increased tension of ciliary muscles after the ICL operation, whereas there were no HFC changes after LASIK surgery. In addition, the vault of the ICL was correlated to postoperative HFC in the ICL group.

Based on previous study [16], the accommodation of a near target in a myopic patient is less than an emmetrope, which could lead to lower activity of the ciliary muscles and longer preservation of accommodation. Thus, after refractive correction, AMP or accommodative function may be improved. However, due to the dysfunction of the ciliary body, zonular fibers, or lens, this improvement may be not observed in patients over 30 years [16]. Moreover, as shown in Prakash’s study [17], although there was an improvement in accommodation in the early period after LAISK, the accommodation could stabilize and approximate the preoperative state at 3 months after the operation. These studies also showed that the AMP could be similar to the preoperative baseline after 3 months of LASIK. In our study, the AMP and the accommodative lag after LASIK may have no identical changes, which was agreement with previous work [4]. In the previous study, Liu et al. [4] also found that there were no changes in the AMP and HFC after LASIK surgery, and they suggested that the LASIK produced no significant effect on accommodation.

Generally, ICL has been considered to maintain the accommodation of eyes because its anterior vault and uncontacted crystalline lens design [18]. Meanwhile, the optic of the ICL needs to be secured in the ciliary sulcus, and there is a possibility that the ICL lens or its footplates may influence the ciliary muscle or tissues around the sulcus. Sheng et al. [19] found that as in the non-accommodative state, more than 53.7% of eyes with footplates rested outside the ciliary sulcus using ultrasound bio-microscopy. They also found that when in an accommodative state, the position of the ciliary-sulcus outside-resting footplate moved closer to the ciliary body or even the zonules. As shown in publications from Kamiya et al. [12], a transient decline of the accommodative amplitude in the early period after ICL implantation was found, and they hypothesized that due to impaction from ICL fixation on the ciliary muscles, the ICL may cause transient dysfunction of the ciliary muscles even if the crystalline lens remained untouched. They did not analyze and evaluate the function of the ciliary muscles directly.

Compared to previous studies on ICL, there was a contradictory outcome related to subjective accommodative function. As shown in Tang’s [20] and Kamiya’s [12] publish, there was a decline of AMP after ICL implantation, while Cheng et al. [11] found that 1 month after ICL surgery, the accommodative function was significantly enhanced, resulting in an increase in AMP, near point convergence, and accommodation facility. Our outcomes showed that the objective amplitude of accommodation had no significant changes after ICL implantation. This discrepancy may be related to different refractive characteristics. As showed in Wan’s study [21], there were different recovery reactions in terms of accommodation when treating myopia with different degrees after ICL surgery. Patients with high myopia may display more obviously accommodative changes after ICL correction. As shown in our study, only eyes with myopia ≤ − 8.5 D were recruited, hence the accommodative function may cause a non-significant change after ICL implantation. Moreover, during the accommodative reaction in the eye with the ICL lens, except when the lens power changes for a specific distance, there were other biometric changes (e.g., in the vault and pupil size) occurring [22, 23]. The power of the eye may be different from expected if the optic eye system cannot remain static [24].

The MFs reflect the influence of the constraints set by the physiological components of the basic mechanism of accommodation; while, it is still unclear what role the MFs play in accommodation. However, one aspect seems clear: the HFC elements of the MFs are not under neurological control and were less dependent on the stimuli conditions (i.e., pupil diameter) [25]. Previous research demonstrated that there was only a certain correlation between HOA and LFC, while, not apparent in the HFC [26]. As shown in our study, there were no significant correlations between HFC and the change of ocular total HOA in either the ICL or the LASIK group.

In a state with ciliary muscle tension, a small accommodative stimulus could cause a large fluctuation and lead to an increased HFC [27]. It should be noted that the HFC may be used to reflect the function of the ciliary muscle. Our study found that there were significant differences in the objective MFs outcome between the ICL and LASIK surgery. This indicated that the HFC of the MFs increased significantly; whereas, there was no change after LASIK surgery. The impaction from ICL fixation may cause transient dysfunction of the ciliary muscles compared to well-balanced LASIK for myopic correction. We further indicated that the ICL lens may produce a reversed force to the ciliary body upon which it was rested and this may increase the tension of the ciliary muscle, resulting in increased HFC. Meanwhile, due to the soft character of the material and the appropriate vault of the ICL lens, the morphology change of the crystalline lens during the maximum and minimum accommodative states would not be impacted, resulting in unchanged AMP. Our study found that there was a slightly positive correlation between HFC and vault; that is, the higher the vault was, the greater the HFC was. This is in accordance with the suggestion above: that ICL with a higher vault may produce a greater reversed force to the ciliary body, which may cause increased HFC compared to ICL with a low vault. In eyes with preoperative MRSE of less than − 8.50 D, there were no significant difference between ICL (− 0.11 D) and LASIK (− 0.09 D) at 3 months after operation in our study, and this refractive outcomes were simialr as previous study [28]. Moreover, in our study, we had found no relationship between MRSE and HFC in both ICL and LASIK groups. Previous literatures show that the degree of myopia is positively correlated with the amount of accommodative HFC [27]. While the effects were influenced by the age and myopic-progression status [25]. In our study, only patietnts with stable myopic progression statues, and their age were older than 18 years were recruited. Then, the HFC may had no significantly changing in LASIK group.

Based on a study within 4 years follow-up, the lense thickness would gradually increase after 12 months of ICL implantation and turn less remarkbale, and they also found that there were a negative raletionship between vault and lens thickness [29]. Richdale et al. foud that accommodation were correlated with lens thickness [30]. So in a long follow-up, the lens rise could be a reason that affacted accommodation after ICL operation.

There were some limitations in our study. First, we observed accommodation and micro-fluctuation only within 3 months follow-up postoperatively. Over this time period, there will be some adaptions. As shown by Kamiya et al. [12], the postoperative accommodative function was impaired in early follow-up after surgery, then recovered gradually. Thus, studies with a longer follow-up period are needed in future work. Second, in this study, we only assessed the MFs outcomes, which reflected the function of the ciliary muscles. A greater value of the HFC of accommodative micro-fluctuations was associated with thinner ciliary bodies using optical coherence tomography [27]. Thus, more measurements containing ciliary muscles or ciliary zonules would be helpful to validate and clarify the structural differences under accommodative reaction in our future work. In addition, we did not objectively evaluate visual discomfort in the patients, and this discomfort when near-working might be negligible in some patients. It would be better if visual discomfort was evaluated with a questionnaire in future work [31].

## Conclusions

ICL (V4c) implantation and LASIK can obtain similar amplitude of accommodation and accommodative lag for myopic correction. Compared to LASIK surgery, the HFC of micro-fluctuations, which reflect tension of the ciliary muscle, increased significantly after 3 months of the ICL implantation, and had a positive correlation with the vault. This supports the view that the ICL lens may produce a reversed force to the ciliary body where it rested on, increasing the tension of the ciliary muscle. Future study with a longer post-operative follow-up time and more structural measurements would be helpful to elucidate the mechanism.

## Data Availability

The datasets used and/or analyzed during the current study available from the corresponding author on reasonable request.

## Abbreviations

ICL: Implantable collamer lens
LASIK: Laser in situ keratomileusis
MFs: Accommodative micro-fluctuation
AMP: Amplitude of accommodation
HOAs: High-order aberrations
HFC: High-frequency component
